# Predictors of CRC Stage at Diagnosis among Male and Female Adults Participating in a Prospective Cohort Study: Findings from Alberta’s Tomorrow Project

**DOI:** 10.3390/curroncol28060414

**Published:** 2021-11-23

**Authors:** Monica Ghebrial, Michelle L. Aktary, Qinggang Wang, John J. Spinelli, Lorraine Shack, Paula J. Robson, Karen A. Kopciuk

**Affiliations:** 1 Cumming School of Medicine, University of Calgary, Calgary, AB T2N 4N1, Canada; monica.ghebrial1@ucalgary.ca; 2 Faculty of Kinesiology, University of Calgary, Calgary, AB T2N 1N4, Canada; michelle.aktary@ucalgary.ca; 3 Cancer Epidemiology and Prevention Research, Cancer Care Alberta, Alberta Health Services, Calgary, AB T2S 3C3, Canada; Qinggang.Wang@albertahealthservices.ca; 4 School of Population and Public Health, University of British Columbia, Vancouver, BC V6T 1Z3, Canada; jspinelli@bccrc.ca; 5 Population Oncology, BC Cancer, Vancouver, BC V5Z 1L3, Canada; 6 Cancer Surveillance and Reporting, Alberta Health Services, Calgary, AB T2S 3C3, Canada; lorraine.shack@albertahealthservices.ca; 7 Department of Agricultural, Food and Nutritional Science and School of Public Health, University of Alberta, Edmonton, AB T6G 2P5, Canada; paula.robson@albertahealthservices.ca; 8 Cancer Care Alberta and Cancer Strategic Clinical Network, Alberta Health Services, Edmonton, AB T5J 3H1, Canada; 9 Departments of Oncology, Community Health Sciences and Mathematics and Statistics, University of Calgary, Calgary, AB T2N 4N2, Canada

**Keywords:** colorectal cancer, stage at diagnosis, Alberta’s Tomorrow Project, Canada, social factors, reproductive factors, sex-specific factors

## Abstract

Colorectal cancer (CRC) is a leading cause of morbidity and mortality in Canada. CRC screening and other factors associated with early-stage disease can improve CRC treatment efficacy and survival. This study examined factors associated with CRC stage at diagnosis among male and female adults using data from a large prospective cohort study in Alberta, Canada. Baseline data were obtained from healthy adults aged 35–69 years participating in Alberta’s Tomorrow Project. Factors associated with CRC stage at diagnosis were evaluated using Partial Proportional Odds models. Analyses were stratified to examine sex-specific associations. A total of 267 participants (128 males and 139 females) developed CRC over the study period. Among participants, 43.0% of males and 43.2% of females were diagnosed with late-stage CRC. Social support, having children, and caffeine intake were predictors of CRC stage at diagnosis among males, while family history of CRC, pregnancy, hysterectomy, menopausal hormone therapy, lifetime number of Pap tests, and household physical activity were predictive of CRC stage at diagnosis among females. These findings highlight the importance of sex differences in susceptibility to advanced CRC diagnosis and can help inform targets for cancer prevention programs to effectively reduce advanced CRC and thus improve survival.

## 1. Introduction

Colorectal cancer (CRC) is the third most common cancer in Canada and the second leading cause of cancer-attributable deaths [1,2]. Data from Canada [1,3] and other developed nations [4,5] indicate that CRC incidence and mortality are higher among males than females. In Canada, age-standardized incidence rates were estimated at 71.7 per 100,000 for males and 50.9 per 100,000 for females [1]. Similarly, in Alberta, Canada, age-standardized incidence rates among males and females were estimated at 62.8 per 100,000 and 45.1 per 100,000, respectively [6]. Coinciding with the aging population, it is expected that one in fourteen males and one in sixteen females in Canada will develop CRC in their lifetime [2].

CRC screening programs and early detection reduce CRC incidence and mortality [7,8,9,10]. Notably, CRC screening may help diagnose CRC at earlier stages, thereby improving a patient’s chance of survival [10,11,12,13]. Cancer staging identifies the severity of cancer at diagnosis according to the size of the tumour, and whether and to what extent it has metastasized to surrounding tissues. Staging ranges from stage I (small and contained) to stage IV (metastasized to other tissues) [10]. In Canada, the net 5-year relative survival of stage IV colon and rectal cancers is estimated at 11% and 12%, respectively, whereas the 5-year survival of stage I colon and rectal cancers is 92% and 87%, respectively [10]. Despite organized CRC screening programs across Canada, in 2015, 49% of CRC diagnoses were stage III and IV [10]. Similarly, in Alberta, 28.6% and 22.0% of all new CRC cases were stage III and IV, respectively [10]. Low population-level participation in screening programs and diagnosis in those who are ineligible for screening may help explain the greater burden of advanced-stage diagnoses.

In addition to low screening participation contributing to later-stage CRC diagnosis, several other important sociodemographic characteristics (e.g., sex, age, race/ethnicity, socioeconomic disadvantage) [10,14,15,16,17,18,19,20,21], and health-related (e.g., family or personal history of polyps or CRC) [14] and psychosocial (e.g., marital status) [15,19,22,23,24,25] factors have been shown to influence stage at diagnosis. For instance, while studies have shown higher CRC incidence and mortality among males, some evidence suggests that females are at greater risk of late-stage diagnoses [16,26,27,28]. The higher risk of advanced CRC among females may be in part due to their greater susceptibility to developing right-sided CRC (i.e., in the proximal colon) [29,30]. Right-sided CRC is less likely to be detected through regular screening methods and typically presents with weaker signs and symptoms (e.g., unrecognized bleeding) than left-sided CRC [29,30]. As a result, right-sided CRC is generally diagnosed at a later stage than left-sided CRC [29,30].

Studies have demonstrated that younger adults are more likely to be diagnosed with late-stage CRC [10,14,15,16,31]. The higher rates among adults under 50 years may be in part due to CRC screening programs beginning at age 50 years for average-risk individuals [32,33,34], potentially delaying diagnoses among younger adults [14]. Moreover, younger adults who present with common CRC symptoms such as abdominal pain and blood in the stool may not be first suspected of having CRC, resulting in longer work-up and delayed diagnosis [31]. Individual- and area-level socioeconomic deprivation have also been associated with later-stage CRC [14,15,16,26]. Advanced CRC rates among low-income populations and ethnic minorities have been observed [16,17,18,26,35], with these disparities primarily attributed to lower screening participation rates [14,36]. Social support has also been associated with stage of CRC diagnosis. Studies have demonstrated that individuals who were married were more likely to be diagnosed with earlier-stage CRC compared to those who were single, separated, or divorced [22,23,24]. Individuals who are married are more likely to have higher household income [24] and health insurance [24], both of which are associated with earlier-stage diagnosis [16,18]. Moreover, spouses may encourage and shape health practices [23,37] such as CRC screening participation [24].

Despite the evidence linking several individual- and environmental-level factors to the stage of CRC diagnosis, some studies have failed to find associations [14,15,38,39]. Differences in study designs, health care systems, sample sizes, and model adjustments likely contribute to the mixed findings among studies. In addition, few studies have examined factors associated with CRC stage at diagnosis in Canada [15,35]. Given that stage at diagnosis is a key prognostic factor for CRC survival [14,40], it is crucial to identify factors predisposing males and females to later-stage diagnoses, and how these factors may differ by sex, to enhance treatment effectiveness and survival. Therefore, the objective of this study is to evaluate sociodemographic characteristics, health- and diet-related factors, and psychosocial factors associated with CRC stage at diagnosis among participants of a large prospective cohort study in Alberta, Canada.

## 2. Materials and Methods

### 2.1. Setting and Participants

Alberta’s Tomorrow Project (ATP) is a prospective cohort study examining health, lifestyle, and psychosocial factors associated with the development and prevention of chronic diseases and cancer among healthy adults living in Alberta, Canada [41]. A total of 55,000 adults aged 35–69 years with no reported history of cancer (excluding non-melanoma skin cancer) were enrolled between 2000 and 2015. History of cancer was cross-checked against the Alberta Cancer Registry to confirm self-reports. Participant recruitment was conducted over two phases: first through random digit dialling using Regional Health Authority boundaries as the sampling frame, and then through a combination of recruitment strategies [42]. Further details of participant recruitment and data collection have been described elsewhere [41].

### 2.2. Data Sources

This study used self-reported data collected from the Health and Lifestyle Questionnaire (HLQ), the Canadian Diet History Questionnaire (CDHQ-I) [43], and the Past-Year Total Physical Activity Questionnaire (PYTPAQ) [44]. Participants completed the surveys at baseline, with a total of 26,890 participants completing all 3 surveys by 2008 [45]. The HLQ assessed sociodemographic characteristics, and psychosocial and health-related variables [46]. The CDHQ-I, a 124-item Food Frequency Questionnaire (FFQ), examined food and nutrient intake over the past year, and the PYTPAQ assessed the type and frequency of physical activity undertaken by participants over the past year [46]. Data from the surveys were subsequently linked to administrative databases using Public Health Numbers for those who consented to data linkage (>99%) [41,42,45].

### 2.3. Outcome

All CRC cases diagnosed as of 18 January 2018 were included in the analysis. The date and stage of cancer diagnosis were ascertained through data linkage with the Alberta Cancer Registry [41,42,45]. The Alberta Cancer Registry records all individual cancer cases and cancer-related deaths across Alberta [47]. The stage of CRC diagnosis was defined by the Tumour, Node, Metastasis staging system [10].

### 2.4. Candidate Explanatory Variables

Candidate explanatory variables were selected based on their association with the development of CRC or stage of CRC diagnosis as informed by the literature, including sociodemographic characteristics (e.g., age, sex, total household income), health- (e.g., cancer screening, family history of CRC) and diet-related (e.g., nutrient intake) factors, and psychosocial factors (e.g., stress, social support).

### 2.5. Statistical Analysis

Descriptive data are presented as median and interquartile range for continuous variables, and as counts and percentages for categorical variables. Differences in variables across CRC stages (i.e., I, II, III/IV) were examined using F-tests for continuous variables and chi-square tests for categorical variables. In a preliminary data assessment, binary variables with low frequencies (i.e., <5) were removed, and categorical variables were collapsed to increase subgroup sizes (i.e., ≥5). Missing responses in continuous variables were imputed using mean value replacement under a missing at random assumption, and missing categorical variables replaced by reference group.

Associations between each candidate explanatory variable and stage at CRC diagnosis were evaluated in bivariate Partial Proportional Odds (PPO) ordinal response models. Variables with a *p*-value < 0.2 were included in the multivariable PPO models. All variables selected were subsequently evaluated in the multivariable PPO model and were retained if *p* < 0.05. Relevant variables associated with the stage at CRC diagnosis were also selected based on the conditional variable importance measure yielded from a random forest analysis using the R package partykit version 1.2-5. Additional variables known to be associated with the stage of CRC diagnosis (i.e., family history of CRC) and those identified from the random forest were forced into the reduced model. The proportional odds assumption was tested, and where the assumption was violated, the model structure was relaxed, allowing the odds ratios (OR) to differ across the outcome cut points (i.e., PPO model).

Functional forms of the continuous variables (e.g., quadratic terms), influence of outliers, and interactions were examined. Model checking was performed using available methods for the binary logistic setting (Stages I and II versus Stages III + IV) in SAS, because many diagnostic tools were not implemented for the PPO model.

Analyses were subsequently stratified to examine whether associations between identified explanatory variables and CRC stage at diagnosis differed by sex. Analyses were conducted in R (version 4.1.0, R Foundation for Statistical Computing, Vienna, Austria) and SAS version 9.4 (SAS Institute, Cary, NC, USA). Statistical significance was set at *p* < 0.05.

## 3. Results

A total of 267 participants (128 males and 139 females) developed CRC over the study period, with a median time of 7 years (quartile 1 to quartile 3: 4 to 10 years) from baseline data collection to receiving a CRC diagnosis. Participant characteristics for males and females by CRC diagnosis stage are presented in Table 1. Among males, 23.4%, 33.6%, and 43.0% were diagnosed with stage I, stage II, and stage III/IV CRC, respectively. The distribution of CRC stage at diagnosis was similar among females, with 22.3%, 34.5%, and 43.2% diagnosed with stage I, stage II, and stage III/IV CRC, respectively. The average age of participants at baseline was 58 years for both males and females, over 96% of participants were Western European ancestry, and a total of 66.2% of females and 64.1% of males had a college or university degree. Just over half (54.7%) of female participants reported a median total annual household income before taxes of <$50,000/year, while a greater proportion of males reported a median total annual household income before taxes of $50,000–$100,000/year. Among males, 76.6% were married and 24.2% were current smokers. Similarly, 71.2% of females were married and 23.7% smoked. Compared to males, females had significantly lower alcohol and red meat consumption, and the higher self-reported CRC screening among females approached significance (*p* = 0.07).

Given the significant differences between males and females in important CRC risk factors, PPO models were stratified by sex. Upon comparing results from the unstratified and stratified analyses, no common factors between sexes were identified. Therefore, only the stratified results are presented. The results from the stratified PPO analysis are summarized in Table 2. Among males, psychosocial factors, including ‘having someone to prepare your meals when unable’ and ‘having someone to hug’ were significantly associated with earlier stage at diagnosis (OR 0.58, 95% CI 0.33–0.99, *p* = 0.05 and OR 0.37, 95% CI 0.14–1.01, *p* = 0.05, respectively). Likewise, having children was significantly associated with earlier stage at diagnosis when comparing stages III/IV vs. II/I (OR 0.33, 95% CI 0.11–1.00, *p* = 0.05). Total caffeine intake (mg/day) was significantly associated with later stage at diagnosis (OR 1.37, 95% CI 1.06–1.76, *p* = 0.02).

Among females, health-related factors that were associated with earlier stage at diagnosis when comparing stages III/IV & II vs. I included family history of CRC (OR 0.21, 95% CI 0.07–0.66, *p* = 0.01) and previously having a hysterectomy (OR 0.28, 95% CI 0.11–0.74, *p* = 0.01). Number of Papanicolaou (Pap) tests in a lifetime (OR 0.96, 95% CI 1.00–1.01, *p* = 0.05) and history of pregnancy (OR 0.21, 95% CI 0.05–0.87, *p* = 0.03) were also protective against late-stage diagnosis. However, history of menopausal hormone therapy (MHT) was significantly associated with later-stage diagnosis (stages III/IV vs. II/I) (OR 3.04, 95% CI 1.41–6.58, *p* < 0.01). In addition, total household physical activity was significantly associated with later-stage CRC diagnosis (OR 1.01, 95% CI 1.00–1.01, *p* = 0.05).

## 4. Discussion

Cancer stage at diagnosis is an important indicator of treatment effectiveness and survival [10]. Despite organized screening programs across Canada and universal health care coverage, nearly 50% of CRC cases are diagnosed at a late stage (III or IV) [10]. This study examined associations between CRC stage at diagnosis and sociodemographic characteristics, health- and diet- related factors, and psychosocial factors among male and female participants in a prospective cohort study in Alberta, Canada. From 2000 to 2018, 267 participants in ATP were diagnosed with CRC, 43.1% of which were late-stage diagnoses (stage III/IV). The overall distribution of CRC by stage was similar among male and female participants. Whereas some studies have demonstrated that females are more frequently diagnosed at a later stage [16,26,27,28], our findings are comparable to Canadian statistics [10].

The current study found that psychosocial and diet-related factors were predictors of CRC stage at diagnosis among males, while health-related factors were more predictive of CRC stage at diagnosis among females. Among males, social support was inversely associated with CRC stage at diagnosis. Evidence suggests that social support is an important factor in CRC outcomes, including the stage at diagnosis [24,48]. A recent systematic review of social factors associated with CRC outcomes identified a number of studies that showed earlier-stage CRC diagnosis and improved CRC survival among those who were married or living with a partner compared to their single counterparts [24]. The inverse association between social support and CRC stage observed only in males may be partly explained by marital status, as studies have shown that males report more social support from their spouse, while females report drawing more social support from friends and relatives [49]. Furthermore, females are more likely to monitor their spouse’s health practices (e.g., cancer screening, attending physician appointments, and dietary intake) and overall health [50,51].

Having children was also inversely associated with CRC stage at diagnosis among males. The protective effects of parenthood against later-stage CRC may be attributed to higher levels of social support, as having children is closely tied to marital status [52,53]. For instance, Kravdal [52] found that the mortality rate of married males with children was 30% lower than that of males without children and those who were never married. Parenthood may also improve CRC outcomes, as having young children is associated with having a stay-at-home partner (generally the mother [53]) who can aid in scheduling healthcare appointments [23]. However, studies have also demonstrated that having children is protective against cancer mortality, even after controlling for marital status [52]. Thus, the link between having children and reduced cancer risk and mortality may be explained by other protective effects conferred by parenthood. For instance, having children may promote feelings of being valued and connected to others and, thus, encourage more self-care behaviours, including participating in cancer screening [48].

This study found diet-related factors associated with the stage of CRC diagnosis among males. Specifically, caffeine intake was associated with increased risk of developing later-stage CRC. Whereas some studies have demonstrated protective effects of coffee [54,55,56,57], tea [58,59], and caffeine [57] consumption against CRC risk and mortality, others have reported null [56,60,61] or harmful effects on CRC outcomes [62,63]. The role of coffee and caffeine in reducing CRC risk may be through various mechanisms, such as anti-proliferative, antioxidant, and anti-inflammatory effects [55,56,62]. However, coffee also contains compounds with mutagenic effects (e.g., glyoxal [64]) that may induce carcinogenesis [56]. Moreover, some studies have identified significant inverse associations between decaffeinated coffee and CRC risk [54,62], suggesting that the observed benefits of coffee consumption may be attributable to other compounds found in coffee rather than caffeine alone. Some studies have suggested that associations between coffee and caffeine consumption and CRC risk may be sex-specific [65]. Higher coffee consumption is associated with the male sex, along with alcohol consumption [60], smoking, and poorer dietary patterns [62,66]. Therefore, the association between caffeine intake and advanced CRC stage at diagnosis in males observed in this study may be due to residual confounding [67,68].

This study identified important health-related factors associated with stage of CRC diagnosis in females. MHT was associated with increased risk of later stage CRC, while history of pregnancy or hysterectomy, lifetime number of Pap tests (cervical cancer screening), and family history of CRC were associated with decreased risk of late-stage CRC. The significant association between MHT and the increased risk of later-stage CRC identified in this study aligns with findings from a randomized controlled trial demonstrating that women on MHT who developed CRC were diagnosed at a later stage than those on the placebo [69]. Whereas a small number of studies have demonstrated associations between MHT usage and later-stage CRC [69,70] and CRC risk and mortality [71,72], study findings have been inconsistent [70,73,74,75,76,77,78,79,80,81,82].

The protective effects of MHT against CRC among females can be partly attributed to the role of oestrogen and the expression of intracellular oestrogen receptors (ER) on inhibiting CRC cell growth [83,84]. Two predominant ER involved in colorectal tumorigenesis include ER-beta and ER-alpha, and their expression may be influenced by timing of MHT initiation. In the pre-cancerous stages of CRC development, exogenous oestrogen activates ER-beta, inhibiting the growth of cancer cells and stimulating apoptosis [85]. However, in the later stages of CRC, the expression of ER-beta is reduced and ER-alpha increases [85]. Exogenous oestrogen then activates ER-alpha, promoting the metastasis of CRC cells [85]. Therefore, it is possible that females in this study began MHT in the later stages of CRC initiation and progression, increasing the risk of advanced CRC.

History of pregnancy and hysterectomy were associated with reduced risk of late-stage CRC diagnosis. Several studies [52,76,86], but not all [81,87,88,89,90], have shown reductions in CRC risk and mortality in females with previous pregnancies compared to their nulliparous counterparts. The protective effects of pregnancy may be due to the higher levels of oestradiol over the course of the pregnancy, and the subsequent effect of oestrogen on reducing bile acid synthesis and insulin-like growth factors [91,92], resulting in reduced risk of carcinogenesis [72,77]. Moreover, investigations of the associations between hysterectomy and CRC risk have either found no associations [78,86] or an increase in CRC risk [92]. A meta-analysis showed a 24% increased risk of CRC for females who had undergone a hysterectomy compared to those who had not undergone surgery [92]. The reduced oestrogen and progesterone levels following a hysterectomy are thought to be involved in increasing CRC risk [92,93].

Associations between reproductive factors and CRC stage at diagnosis may also be related to sociocultural factors. Females are more likely than males to regularly visit their physician, even after adjusting for reproductive health care visits [94,95]. Indeed, Canadian data suggest that 85.5% of Canadians (85.1% of Albertans) have a regular family physician, with physician attachment higher among females than males [96]. Regular physician visits, increased exposure to cancer screening, and physician-made CRC screening recommendations have shown to improve CRC screening uptake and participation [94,97,98,99,100,101]. Moreover, family history of CRC is an important risk factor for CRC [14]. As a result, females with a family history of CRC may be more likely to undergo CRC screening [102].

Higher levels of household physical activity (e.g., housework) were associated with later stage CRC diagnosis among females. Contrary to our findings, evidence has demonstrated that higher levels of physical activity are associated with reduced CRC risk [61,103,104,105,106]. Moreover, a recent meta-analysis found a significant inverse association between household physical activity and CRC risk among females (relative risk (RR) 0.78, 95% CI 0.69–0.88) but not males (RR 1.04, 95% CI 0.84–1.30) [105]. However, studies have demonstrated that females tend to spend more time in occupational and household activities than recreational activities [106,107,108,109,110], which may create time barriers and thus potentially delay physician visits or cancer screening.

Our study has several strengths. First, this study examined associations between several important sociodemographic characteristics, health- and diet-related factors, and psychosocial factors and CRC stage at diagnosis using data from a large population-level cohort. Second, the prospective cohort study design allowed the assessment of CRC risk factors prior to cancer diagnosis, thus minimizing recall bias [111]. Third, we collected data using valid and reliable surveys and assessment tools [44,112]. However, the study results should be interpreted considering some limitations. Data for this study were collected using self-reported measures, and thus are subject to social desirability bias [113]. Social desirability bias may be of particular concern with measures such as self-reported physical activity [113,114], dietary intake [115], and less socially acceptable practices, such as smoking and excess alcohol consumption [116]. In addition, dietary intake was examined using an FFQ. Whereas FFQs are recommended dietary assessment tools, they are subject to recall bias and measurement error [117]. Our study had limited ethnic/racial diversity, with over 90% of participants of European descent, thus limiting generalizability of these results. However, according to statistics from 2001 (when these ATP data were being collected), close to 90% of individuals living in Alberta were White [118]. Therefore, the study sample is representative of the Alberta population in the early 2000s. Moreover, the surveys did not allow for detailed assessment of MHT. Given the complexity of hormonal and physiological processes, the ability to stratify our analysis by MHT type, timing, or duration may have provided further insight into the observed association. However, with only 139 cases of CRC detected in females over the study period, our study was not powered to further stratify our analyses. Finally, while our analyses controlled for important measured confounders, we cannot rule out the possibility of residual confounding.

## 5. Conclusions

This study found several novel factors associated with CRC stage at diagnosis among males and females. Social support and caffeine intake were important predictors of CRC stage among males, while reproductive and hormonal factors and household physical activity predicted CRC stage at diagnosis among females. These findings highlight important sex-specific differences in susceptibility to advanced CRC. Whereas regular CRC screening remains fundamental in the detection of earlier-stage CRC, identifying other important factors associated with CRC stage at diagnosis, particularly those specific to males and females, can help inform targets for cancer prevention programs to effectively reduce advanced CRC and thus greatly improve CRC survival.

## Figures and Tables

**Table 1:** Description of the study population by sex and cancer diagnosis stage.

Characteristic *N* (%)	Male (*N* = 128)				Female (*N* = 139)			
	Stage I (*N* = 30)	Stage II (*N* = 43)	Stages III & IV (*N* = 55)	Total (*N* = 128)	Stage I (*N* = 31)	Stage II (*N* = 48)	Stages III & IV (*N* = 60)	Total (*N* = 139)
Age at baseline (years)								
<60	18 (14.8)	24 (18.7)	37 (28.9)	79 (61.7)	16 (11.5)	25 (18.0)	40 (28.8)	81 (58.2)
60+	12 (9.8)	19 (14.8)	18 (14.1)	49 (38.3)	15 (10.8)	23 (16.6)	20 (14.4)	58 (41.7)
Age at diagnosis (years)								
<60	-	-	21 (16.4)	37 (28.9)	-	16 (11.5)	21 (15.1)	45 (32.4)
60+	22 (17.2)	35 (27.3)	34 (26.6)	81 (71.1)	23(16.6)	32 (23.1)	39 (28.1)	94 (67.6)
Education								
High school diploma	11 (8.6)	14 (10.9)	21 (16.4)	46 (35.9)	14 (10.1)	15 (10.8)	18 (13.0)	47 (33.8)
Post-secondary	19 (14.8)	29 (22.7)	34 (26.6)	82 (64.1)	17 (12.2)	33 (23.7)	42 (30.2)	90 (66.2)
Married or common in-law								
Yes	29 (22.7)	29 (22.7)	40 (31.3)	98 (76.6) **	22 (15.8)	34 (24.5)	43 (30.9)	99 (71.2)
No	1 (0.8)	14 (10.9)	15 (11.7)	30 (23.4)	9 (6.5)	14 (10.1)	17 (12.2)	40 (28.8)
Employment								
Yes	23 (18.0)	27 (21.1)	36 (28.1)	86 (67.2)	15 (10.8)	27 (19.4)	28 (20.1)	70 (50.4)
No	7 (5.5)	16 (12.5)	19 (14.8)	42 (32.8)	16 (11.5)	21 (15.1)	32 (23.0)	69 (49.6)
Household Income ($)								
<50 K	7 (5.5)	20 (15.6)	16 (12.5)	43 (33.6)	21 (15.1)	25 (18.0)	30 (21.6)	76 (54.7)
50–100 K	15 (11.7)	13 (10.2)	24 (18.8)	52 (40.6)	7 (5.0)	17 (12.2)	24 (17.3)	48 (34.5)
>100 K	8 (6.3)	10 (7.8)	15 (11.7)	33 (25.8)	3 (2.2)	6 (4.3)	6 (4.3)	15 (10.8)
Geography residence ^£^								
Rural	12 (9.4)	15 (11.7)	11 (8.6)	38 (29.7)	9 (6.5)	15 (10.8)	20 (14.4)	44 (31.7)
Urban	18 (14.1)	28 (21.9)	44 (34.4)	90 (70.3)	22 (15.8)	33 (23.7)	40 (28.8)	95 (68.3)
First degree colorectal cancer family history ^£^								
Yes	4 (3.1)	6 (4.7)	9 (7.0)	19 (14.8)	8 (5.7)	3 (2.2)	10 (7.2)	21 (15.1)
No	26 (20.3)	37 (28.9)	46 (35.9)	109 (85.2)	23 (16.6)	45 (32.4)	50 (36.0)	118 (84.9)
Ever had a digital rectal exam								
Yes	24 (18.8)	33 (25.8)	44 (34.4)	101 (78.9)	18 (13.0)	25 (18.0)	28 (20.1)	71 (51.1)
No	6 (4.7)	10 (7.8)	11 (8.6)	27 (21.1)	13 (9.4)	23 (16.6)	32 (23.0)	68 (48.9)
Ever had a blood stool test								
Yes	11 (8.6)	9 (7.0)	18 (14.1)	38 (29.7)	10 (7.2)	21 (15.1)	25 (18.0)	56 (40.2)
No	19 (14.8)	34 (26.6)	37 (28.9)	90 (70.3)	21 (15.1)	27 (19.4)	35 (25.2)	83 (59.7)
Have ever had a sigmoidoscopy or colonoscopy done								
Yes	2 (1.6)	7 (5.5)	10 (7.8)	19 (14.8)	8 (5.8)	12 (8.6)	13 (9.4)	33 (23.7)
No	28 (21.9)	36 (28.2)	45 (35.2)	109 (85.2)	23 (16.6)	36 (25.9)	47 (33.8)	106 (76.3)
Have ever had a PSA blood test								
Yes	12 (9.4)	21 (16.4)	20 (15.6)	53 (41.4)				
No	18 (14.1)	21 (16.4)	35 (27.3)	74 (57.8)				
Have ever been pregnant								
Yes					30 (21.6)	46 (33.1)	52 (37.4)	128 (92.1)
No					1 (0.8)	2 (1.4)	8 (5.8)	11 (7.9)
Have ever had a hysterectomy								
Yes					15 (10.8)	12 (8.6)	18 (13.0)	45 (32.4)
No					16 (11.5)	36 (25.9)	42 (30.2)	94 (67.6)
Female Hormones used for menopause **								
Yes					17 (12.2)	12 (8.6)	32 (23.0)	61 (43.9)
No					14 (10.1)	36 (25.9)	28 (20.1)	78 (56.1)
Have ever had Pap smear test								
Yes					31 (22.3)	45 (32.4)	60 (43.2)	136 (97.8)
No					0 (0)	3 (2.2)	0 (0)	3 (2.2)
Have ever had mammogram test								
Yes					28 (20.1)	42 (30.2)	51 (36.7)	121 (87.1)
No					3 (2.2)	6 (4.3)	9 (6.5)	18 (13.0)
Type of smoker								
Non smoker	10 (7.8)	14 (10.9)	11 (8.6)	35 (27.3)	12 (8.6)	12 (8.6)	26 (18.7)	50 (36.0)
Past smoker	16 (12.5)	18 (14.1)	28 (21.9)	62 (48.4)	13 (9.4)	21 (15.1)	22 (15.8)	56 (40.3)
Current smoker	4 (3.1)	11 (8.6)	16 (12.5)	31 (24.2)	6 (4.3)	15 (10.8)	12 (8.6)	33 (23.7)
Spend in the sun 11 a.m.–4 p.m. in June –August (hours)								
<1	23 (18.0)	33 (25.8)	37 (28.9)	93 (72.7)	15 (10.8)	27 (19.4)	31 (22.3)	73 (52.5)
≥1	7 (5.5)	10 (7.8)	18 (14.1)	35 (27.3)	16 (11.5)	21 (15.1)	29 (20.9)	66 (47.5)
Any current stressful situations								
None	19 (14.8)	27 (21.1)	29 (22.7)	75 (58.6)	19 (13.7)	23 (16.6)	40 (28.8)	82 (59.0)
≥1	11 (8.6)	16 (12.5)	26 (20.3)	53 (41.4)	12 (8.6)	25 (18.0)	20 (14.4)	57 (41.0)
Any children								
Yes	26 (20.3)	40 (31.3)	44 (34.4)	110 (85.9)	29 (20.9)	46 (33.1)	53 (38.1)	128 (92.1)
No	4 (3.1)	3 (2.3)	11 (8.6)	18 (14.1)	2 (1.4)	2 (1.4)	7 (5.0)	11 (7.9)
Too much is expected of you by others								
Yes	9 (7.0)	12 (9.4)	17 (13.3)	38 (29.7)	12 (8.6)	18 (13.0)	15 (10.8)	45 (32.4)
No	21 (16.4)	31 (24.2)	38 (29.7)	90 (70.3)	19 (13.7)	30 (21.6)	45 (32.4)	94 (67.6)
	**Mean (SD)**							
	**Male**				**Female**			
Age at baseline (years)	56.3 (7.8)	58.3 (7.1)	54.7 (8.7)	56.3 (8.1)	57.5 (8.8)	56.0 (9.5)	55.4 (8.9)	56.1 (9.1)
Distance to accessible health centre by a vehicle (minutes)	11.6 (11.8)	13.1 (12.3)	9.9 (8.0)	11.4 (10.6)	8.3 (7.3)	8.4 (7.9)	10.2 (10.3)	9.1 (8.9)
Total body mass index (BMI)	28.9 (5.6)	30.0 (5.1)	30.7 (5.7)	30.0 (5.5)	28.6 (6.0)	28.4 (5.2)	27.6 (5.3)	28.1 (5.4)
Total physical activity (Met-hours/week)	164.5 (81.5)	136.3 (74.1)	146.4 (51.5)	147.3 (75.0)	147.4 (89.8)	152.5 (79.8)	142.9 (68.8)	147.2 (77.2)
Total recreational activity (Met-hours/week)	34.8 (31.3)	24.8 (21.7)	26.9 (30.6)	28.1 (28.1)	17.2 (15.0)	17.1 (14.9)	18.7 (18.0)	17.8 (16.2)
Total household activity (Met-hours/week)	35.1 (29.1)	38.3 (33.7)	39.3 (26.6)	38.0 (28.1)	59.4 (40.5)	70.6 (50.9)	80.4 (53.6)	72.3 (50.3)
Total dietary caloric intake (kcal/day)	2239 (1035)	2235 (980)	2370 (1053)	2294 (1019)	1560 (578)	1674 (756)	1513 (610)	1579 (657)
Total protein intake (g/day)	87 (39)	79 (31)	89 (41)	85 (38)	61 (24)	64 (26)	58 (21)	61 (24)
Total caffeine intake (mg/day)	490 (435)	676 (582)	630 (443)	609 (492)	451 (380)	476 (346)	448 (405)	457 (381)
	**Median (Q1** **–** **Q3)**							
	**Male**				**Female**			
Comorbidity index ^£^	0 (0–1)	1 (0–1)	0 (0–1)	1 (0–1)	1 (0–1)	1 (0–1)	0 (0–1)	1 (0–1)
Number of PSA tests in lifetime	0 (0–1)	1 (0–2)	0 (0–2)	0 (0–2)				
Number of Pap smear tests in lifetime					12 (8–25)	16 (9–20)	11 (10–19)	12 (10–20)
Number of mammograms in lifetime ^§^					5 (3–10)	4 (1–8)	5 (2–8)	4 (2–9)
Social support: someone to hug: scale 1–5	5 (5–5)	4 (3–5)	5 (4–5)	5 (4–5) *	5 (4–5)	5 (4–5)	5 (4–5)	5 (4–5)
Social support: someone to prepare your meals when you were unable to do it yourself: scale 1–5	5 (4–5)	4 (4–5)	4 (3–5)	4 (4–5) *	4 (3–5)	4 (3–5)	4 (3–5)	4 (3–5)
Total alcohol intake (g/day)	13 (2–23)	5 (1–15)	4 (2–25)	5 (2–21)	1 (0–6)	2 (1–7)	2 (0–8)	2 (0–7)
Daily fruit and vegetable intake (servings/day)	4 (3–6)	3 (2–6)	3 (2–6)	3 (2–6)	4 (3–7)	4 (2–6)	4 (3–7)	4 (3–7)
Daily red meat intake (servings/day)	3 (2–4)	2 (1–3)	2 (1–4)	2 (1–4)	1 (1–2)	1 (1–2)	1 (1–2)	1 (1–2)

Statistical significance within each gender at * *p* < 0.05, ** *p* < 0.01. ^§^ The number of mammogram tests in lifetime is only for these who reported to have mammogram test. ^£^ These variables are derived from the Alberta Cancer Registry, Administration data. Elixhauser Comorbidity Index: range (0–7). ‘-’ indicates number for age was <10 so cell entry suppressed.

**Table 2:** Predictors of colorectal cancer diagnosis at different stages from the Partial Proportional Odds Regression Model.

Predictor Variable		Males (*N* = 128)			Females (*N* = 139)	
	Cancer Stage (PPO)	ORs (95% CI)	*p* Value	Cancer Stage (PPO)	ORs (95% CI)	*p* Value
Colorectal cancer family history						
Yes vs. No		1.24 (0.48–3.24)	0.66	III/IV vs. II & I	1.65 (0.58–4.74)	0.35
				III/IV & II vs. I	0.21 (0.07–0.66)	0.01
Have ever been pregnant						
Yes vs. No					0.21 (0.05–0.87)	0.03
Have ever had a hysterectomy						
Yes vs. No				III/IV vs. II & I	0.58 (0.26–1.33)	0.20
				III/IV & II vs. I	0.28 (0.11–0.74)	0.01
Female Hormones used for menopause						
Yes vs. No				III/IV vs. II & I	3.04 (1.41–6.58)	<0.01
				III/IV & II vs. I	0.69 (0.28–1.73)	0.43
Number of Pap tests in lifetime					0.96 (0.93–1.00)	0.04
Total household activity (Met-hours/week)					1.01 (1.00–1.01)	0.05
Total household activity (Met-hours/week)						
<40					1.0	
40–60					4.08 (1.54–10.82)	<0.01
>60					3.53 (1.49–8.37)	<0.01
Any children						
Yes vs. No	III/IV vs. II & I	0.33 (0.11–1.00)	0.05			
	III/IV & II vs. I	1.57 (0.39–6.33)	0.53			
Social support: someone to hug: scale 1–5						
	III/IV vs. II & I	1.56 (0.90–2.71)	0.12			
	III/IV & II vs. I	0.37 (0.14–1.01)	0.05			
Social support: someone to prepare your meals when you were unable to do it yourself: scale 1–5						
	III/IV vs. II & I	0.58 (0.33–0.99)	0.05			
	III/IV & II vs. I	0.90 (0.40–2.01)	0.80			
Total caffeine intake (mg/day) at log scale						
	III/IV vs. II & I	1.22 (0.92–1.63)	0.18			
	III/IV & II vs. I	1.37 (1.06–1.76)	0.02			

Note that: Cancer stage III/IV means stage III & IV. Whenever there is entry for the cancer stage column, it means the results are from a partial proportional odds model. Otherwise, it is the proportional odds model where coefficients are same for stages III/IV vs. II & I and III/IV & II vs. I.

## Data Availability

Access to individual-level data is available in accordance with the Health Information Act of Alberta and Alberta’s Tomorrow Project (ATP). Access Guidelines at https://myatpresearch.ca (accessed on 18 January 2018).

## References

[1] (2019). Canadian Cancer Statistics. https://cancer.ca/en/research/cancer-statistics/canadian-cancer-statistics.

[2] Brenner D.R., Weir H.K., Demers A.A., Ellison L.F., Louzado C., Shaw A., Turner D., Woods R.R., Smith L.M., Canadian Cancer Statistics Advisory Committee (2020). Projected Estimates of Cancer in Canada in 2020. CMAJ.

[3] Gao R.N., Neutel C.I., Wai E. (2008). Gender Differences in Colorectal Cancer Incidence, Mortality, Hospitalizations and Surgical Procedures in Canada. J. Public Health (Oxf).

[4] Ferlay J., Shin H.R., Bray F., Forman D., Mathers C., Parkin D.M. (2010). Estimates of worldwide burden of cancer in 2008: GLOBOCAN 2008. Int. J. Cancer.

[5] Nguyen S.P., Bent S., Chen Y.H., Terdiman J.P. (2009). Gender as a Risk Factor for Advanced Neoplasia and Colorectal Cancer: A Systematic Review and Meta-Analysis. Clin. Gastroenterol. Hepatol..

[6] Cancer Care Alberta The 2019 Report on Cancer Statistics in Alberta-Colorectal. https://public.tableau.com/profile/cancercontrol.ab#!/vizhome/The2019ReportonCancerStatisticsinAlberta/Highlights?publish=yes.

[7] Leddin D.J., Enns R., Hilsden R., Plourde V., Rabeneck L., Sadowski D.C., Singh H. (2010). Canadian Association of Gastroenterology Position Statement on Screening Individuals at Average Risk for Developing Colorectal Cancer: 2010. Can. J. Gastroenterol..

[8] Schoen R.E., Pinsky P.F., Weissfeld J.L., Yokochi L.A., Church T., Laiyemo A.O., Bresalier R., Andriole G.L., Buys S.S., Crawford D. (2012). Colorectal-Cancer Incidence and Mortality with Screening Flexible Sigmoidoscopy. N. Engl. J. Med..

[9] Hewitson P., Glasziou P., Watson E., Towler B., Irwig L. (2008). Cochrane Systematic Review of Colorectal Cancer Screening Using the Fecal Occult Blood Test (Hemoccult): An Update. Am. J. Gastroenterol..

[10] Canadian Cancer Statistics Advisory Committee (2018). Canadian Cancer Statistics 2018.

[11] Moreno C.C., Mittal P.K., Sullivan P.S., Rutherford R., Staley C.A., Cardona K., Hawk N.N., Dixon W.T., Kitajima H.D., Kang J. (2016). Colorectal Cancer Initial Diagnosis: Screening Colonoscopy, Diagnostic Colonoscopy, or Emergent Surgery, and Tumor Stage and Size at Initial Presentation. Clin. Colorectal Cancer.

[12] Fazio L., Cotterchio M., Manno M., McLaughlin J., Gallinger S. (2005). Association between Colonic Screening, Subject Characteristics, and Stage of Colorectal Cancer. Am. J. Gastroenterol..

[13] Cole S.R., Tucker G.R., Osborne J.M., Byrne S.E., Bampton P.A., Fraser R.J., Young G.P. (2013). Shift to Earlier Stage at Diagnosis as a Consequence of the National Bowel Cancer Screening Program. Med. J. Aust..

[14] Andrew A.S., Parker S., Anderson J.C., Rees J.R., Robinson C., Riddle B., Butterly L.F. (2018). Risk Factors for Diagnosis of Colorectal Cancer at a Late Stage: A Population-Based Study. J. Gen. Intern. Med..

[15] Blair A., Datta G.D. (2020). Associations between Area-Level Deprivation, Rural Residence, Physician Density, Screening Policy and Late-Stage Colorectal Cancer in Canada. Cancer Epidemiol..

[16] Halpern M.T., Pavluck A.L., Ko C.Y., Ward E.M. (2009). Factors Associated with Colon Cancer Stage at Diagnosis. Dig. Dis. Sci..

[17] Haas J.S., Earle C.C., Orav J.E., Brawarsky P., Neville B.A., Williams D.R. (2008). Racial Segregation and Disparities in Cancer Stage for Seniors. J. Gen. Intern. Med..

[18] Askari A., Nachiappan S., Currie A., Latchford A., Stebbing J., Bottle A., Athanasiou T., Faiz O. (2017). The Relationship between Ethnicity, Social Deprivation and Late Presentation of Colorectal Cancer. Cancer Epidemiol..

[19] Lai K.-C., Stotler B.A. (2010). Marital Status and Colon Cancer Stage at Diagnosis. Open Colorectal Cancer J..

[20] White A., Ironmonger L., Steele R.J.C., Ormiston-Smith N., Crawford C., Seims A. (2018). A Review of Sex-Related Differences in Colorectal Cancer Incidence, Screening Uptake, Routes to Diagnosis, Cancer Stage and Survival in the UK. BMC Cancer.

[21] Wu X.C., Chen V.W., Steele B., Ruiz B., Fulton J., Liu L., Carozza S.E., Greenlee R. (2001). Subsite-Specific Incidence Rate and Stage of Disease in Colorectal Cancer by Race, Gender, and Age Group in the United States, 1992–1997. Cancer.

[22] Aizer A.A., Chen M.H., McCarthy E.P., Mendu M.L., Koo S., Wilhite T.J., Graham P.L., Choueiri T.K., Hoffman K.E., Martin N.E. (2013). Marital Status and Survival in Patients with Cancer. J. Clin. Oncol..

[23] Wang L., Wilson S.E., Stewart D.B., Hollenbeak C.S. (2011). Marital Status and Colon Cancer Outcomes in US Surveillance, Epidemiology and End Results registries: Does Marriage Affect Cancer Survival by Gender and Stage?. Cancer Epidemiol..

[24] Coughlin S.S. (2020). Social Determinants of Colorectal Cancer Risk, Stage, and Survival: A Systematic Review. Int. J. Colorectal Dis..

[25] Tromp D.M., Brouha X.D., De Leeuw J.R., Hordijk G.J., Winnubst J.A. (2004). Psychological Factors and Patient Delay in Patients with Head and Neck Cancer. Eur. J. Cancer.

[26] Perdue D.G., Haverkamp D., Perkins C., Daley C.M., Provost E. (2014). Geographic Variation in Colorectal Cancer Incidence and Mortality, Age of Onset, and Stage at Diagnosis among American Indian and Alaska Native People, 1990–2009. Am. J. Public Health.

[27] Woods S.E., Basho S., Engel A. (2006). The Influence of Gender on Colorectal Cancer Stage—The State of Ohio, 1996–2001. J. Women’s Health.

[28] Mandelblatt J., Andrews H., Kao R., Wallace R., Kemer J. (1996). The Late-Stage Diagnosis of Colorectal Cancer-Demographic and Socioeconomic Factors. J. Public Health.

[29] Hansen I.O., Jess P. (2012). Possible Better Long-Term Survival in Left Versus Right-Sided Colon Cancer—A Systematic Review. Dan. Med. J..

[30] Nawa T., Kato J., Kawamoto H., Okada H., Yamamoto H., Kohno H., Endo H., Shiratori Y. (2008). Differences between Right-and Left-Sided Colon Cancer in Patient Characteristics, Cancer Morphology and Histology. J. Gastroenterol. Hepatol..

[31] Chen F.W., Sundaram V., Chew T.A., Ladabaum U. (2017). Advanced-Stage Colorectal Cancer in Persons Younger Than 50 Years Not Associated With Longer Duration of Symptoms or Time to Diagnosis. Clin. Gastroenterol. Hepatol..

[32] Morgan J.W., Cho M.M., Guenzi C.D., Jackson C., Mathur A., Natto Z., Kazanjian K., Tran H., Shavlik D., Lum S.S. (2011). Predictors of Delayed-Stage Colorectal Cancer: Are We Neglecting Critical Demographic Information?. Ann. Epidemiol..

[33] Cancer Care Ontario Colorectal Cancer Screening Recommendations Summary. https://www.cancercareontario.ca/en/guidelines-advice/cancer-continuum/screening/resources-healthcare-providers/colorectal-cancer-screening-summary.

[34] Center for Disease Control (CDC) What Is Colorectal Cancer Screening?. https://www.cdc.gov/cancer/colorectal/basic_info/screening/index.htm#:~:text=Screening%20Guidelines,if%20they%20should%20be%20screened.

[35] Booth C.M., Li G., Zhang-Salomons J., Mackillop W.J. (2010). The Impact of Socioeconomic Status on Stage of Cancer at Diagnosis and Survival: A Population-Based Study in Ontario, Canada. Cancer.

[36] O’Malley A.S., Beaton E., Yabroff K.R., Abramson R., Mandelblatt J. (2004). Patient and Provider Barriers to Colorectal Cancer Screening in the Primary Care Safety-Net. Prev. Med..

[37] Schone B.S., Weinick R.M. (1998). Health-Related Behaviors and the Benefits of Marriage for Elderly Persons. Gerontology.

[38] Schwartz K.L., Crossley-May H., Vigneau F.D., Brown K., Banerjee M. (2003). Race, Socioeconomic Status and Stage at Diagnosis for Five Common Malignancies. Cancer Causes Control.

[39] Frederiksen B.L., Osler M., Harling H., Jorgensen T., Danish Colorectal Cancer Group (2008). Social Inequalities in Stage at Diagnosis of Rectal but Not in Colonic Cancer: A Nationwide Study. Br. J. Cancer.

[40] Nausheen B., Gidron Y., Peveler R., Moss-Morris R. (2009). Social Support and Cancer Progression: A Systematic Review. J. Psychosom. Res..

[41] Robson P.J., Solbak N.M., Haig T.R., Whelan H.K., Vena J.E., Akawung A.K., Rosner W.K., Brenner D.R., Cook L.S., Csizmadi I. (2016). Design, Methods and Demographics from Phase I of Alberta’s Tomorrow Project Cohort: A Prospective Cohort Profile. CMAJ Open.

[42] Ye M., Robson P.J., Eurich D.T., Vena J.E., Xu J.Y., Johnson J.A. (2017). Cohort Profile: Alberta’s Tomorrow Project. Int. J. Epidemiol..

[43] Csizmadi I., Kahle L., Ullman R., Dawe U., Zimmerman T.P., Friedenreich C.M., Bryant H., Subar A.F. (2007). Adaptation and Evaluation of the National Cancer Institute’s Diet History Questionnaire and Nutrient Database for Canadian Populations. Public Health Nutr..

[44] Friedenreich C.M., Courneya K.S., Neilson H.K., Matthews C.E., Willis G., Irwin M., Troiano R., Ballard-Barbash R. (2006). Reliability and Validity of the Past Year Total Physical Activity Questionnaire. Am. J. Epidemiol..

[45] Bryant H., Robson P.J., Ullman R., Friedenreich C., Dawe U. (2006). Population-Based Cohort Development in Alberta, Canada: A Feasibility Study. Chronic Dis. Can..

[46] Alberta’s Tomorrow Project Survey Questions Asked. https://myatpresearch.ca/survey-questions/.

[47] Alberta Health Services Alberta Cancer Registry—Cancer Research & Analytics. https://www.albertahealthservices.ca/cancer/Page17367.aspx.

[48] Kinney A.Y., Bloor L.E., Dudley W.N., Millikan R.C., Marshall E., Martin C., Sandler R.S. (2003). Roles of Religious Involvement and Social Support in the Risk of Colon Cancer Among Blacks and Whites. Am. J. Epidemiol..

[49] Gurung R.A., Taylor S.E., Seeman T.E. (2003). Accounting for Changes in Social Support among Married Older Adults: Insights from the MacArthur Studies of Successful Aging. Psychol. Aging.

[50] Umberson D. (1992). Gender, Marital Status and the Social Control of Health Behavior. Soc. Sci. Med..

[51] Mroz L.W., Robertson S. (2015). Gender Relations and Couple Negotiations of British Men’s Food Practice Changes after Prostate Cancer. Appetite.

[52] Kravdal O. (2003). Children, Family and Cancer Survival in Norway. Int. J. Cancer.

[53] Ross C.E., Mirowsky J., Goldsteen K. (1990). The Impact of the Family on Health: The Decade in Review. J. Marriage Fam..

[54] Sinha R., Cross A.J., Daniel C.R., Graubard B.I., Wu J.W., Hollenbeck A.R., Gunter M.J., Park Y., Freedman N.D. (2012). Caffeinated and Decaffeinated Coffee and Tea Intakes and Risk of Colorectal Cancer in a Large Prospective Study. Am. J. Clin. Nutr..

[55] Mackintosh C., Yuan C., Ou F.S., Zhang S., Niedzwiecki D., Chang I.W., O’Neil B.H., Mullen B.C., Lenz H.J., Blanke C.D. (2020). Association of Coffee Intake With Survival in Patients With Advanced or Metastatic Colorectal Cancer. JAMA Oncol..

[56] Sartini M., Bragazzi N.L., Spagnolo A.M., Schinca E., Ottria G., Dupont C., Cristina M.L. (2019). Coffee Consumption and Risk of Colorectal Cancer: A Systematic Review and Meta-Analysis of Prospective Studies. Nutrients.

[57] Guercio B.J., Sato K., Niedzwiecki D., Ye X., Saltz L.B., Mayer R.J., Mowat R.B., Whittom R., Hantel A., Benson A. (2015). Coffee Intake, Recurrence, and Mortality in Stage III Colon Cancer: Results From CALGB 89803 (Alliance). J. Clin. Oncol..

[58] Yang G., Zheng W., Xiang Y.B., Gao J., Li H.L., Zhang X., Gao Y.T., Shu X.O. (2011). Green Tea Consumption and Colorectal Cancer Risk: A Report from the Shanghai Men’s Health Study. Carcinogenesis.

[59] Yang G., Shu X.O., Li H., Chow W.H., Ji B.T., Zhang X., Gao Y.T., Zheng W. (2007). Prospective Cohort Study of Green Tea Consumption and Colorectal Cancer Risk in Women. Cancer Epidemiol. Biomark. Prev..

[60] Micek A., Gniadek A., Kawalec P., Brzostek T. (2019). Coffee Consumption and Colorectal Cancer Risk: A Dose-Response Meta-analysis on Prospective Cohort studies. Int. J. Food Sci. Nutr..

[61] World Cancer Research Fund, American Institute for Cancer Research (2018). Diet, Nutrition, Physical Activity and Colorectal Cancer.

[62] Um C.Y., McCullough M.L., Guinter M.A., Campbell P.T., Jacobs E.J., Gapstur S.M. (2020). Coffee Consumption and Risk of Colorectal Cancer in the Cancer Prevention Study-II Nutrition Cohort. Cancer Epidemiol..

[63] Simons C.C., Leurs L.J., Weijenberg M.P., Schouten L.J., Goldbohm R.A., van den Brandt P.A. (2010). Fluid Intake and Colorectal Cancer Risk in the Netherlands Cohort Study. Nutr. Cancer.

[64] Nagao M., Fujita Y., Wakabayashi K. (1986). Mutagens in Coffee and Other Beverages. Environ. Health Perspect..

[65] Slattery M.L., West D.W., Robison L.M., French T.K., Ford M.H., Schuman K.L., Sorenson A.W. (1990). Tobacco, Alcohol, Coffee, and Caffeine as Risk Factors for Colon Cancer in a Low-Risk Population. Epidemiology.

[66] Elhadad M.A., Karavasiloglou N., Wulaningsih W., Tsilidis K.K., Tzoulaki I., Patel C.J., Rohrmann S. (2020). Metabolites, Nutrients, and Lifestyle Factors in Relation to Coffee Consumption: An Environment-Wide Association Study. Nutrients.

[67] Johnson C.M., Wei C., Ensor J.E., Smolenski D.J., Amos C.I., Levin B., Berry D.A. (2013). Meta-Analyses of Colorectal Cancer Risk Factors. Cancer Causes Control.

[68] Tung J., Politis C.E., Chadder J., Han J., Niu J., Fung S., Rahal R., Earle C.C. (2018). The North-South and East-West Gradient in Colorectal Cancer Risk: A Look at the Distribution of Modifiable Risk Factors and Incidence across Canada. Curr. Oncol..

[69] Simon M.S., Chlebowski R.T., Wactawski-Wende J., Johnson K.C., Muskovitz A., Kato I., Young A., Hubbell F.A., Prentice R.L. (2012). Estrogen Plus Progestin and Colorectal Cancer Incidence and Mortality. J. Clin. Oncol..

[70] Chlebowski R.T., Wactawski-Wende J., Ritenbaugh C., Hubbell A., Ascensao J., Rodabough R.J., Rosenberg C.A., Taylor V.M., Harris R., Chen C. (2004). Estrogen plus Progestin and Colorectal Cancer in Postmenopausal Women. N. Engl. J. Med..

[71] Simin J., Liu Q., Wang X., Fall K., Williams C., Callens S., Engstrand L., Brusselaers N. (2021). Prediagnostic Use of Estrogen-Only Therapy Is Associated with Improved Colorectal Cancer Survival in Menopausal Women: A Swedish Population-Based Cohort Study. Acta Oncol..

[72] Gunter M.J., Hoover D.R., Yu H., Wassertheil-Smoller S., Rohan T.E., Manson J.E., Howard B.V., Wylie-Rosett J., Anderson G.L., Ho G.Y. (2008). Insulin, Insulin-like Growth Factor-I, Endogenous Estradiol, and Risk of Colorectal Cancer in Postmenopausal Women. Cancer Res..

[73] Morch L.S., Lidegaard O., Keiding N., Lokkegaard E., Kjaer S.K. (2016). The Influence of Hormone Therapies on Colon and Rectal cancer. Eur. J. Epidemiol..

[74] Hannaford P., Elliott A. (2005). Use of Exogenous Hormones by Women and Colorectal Cancer: Evidence from the Royal College of General Practitioners’ Oral Contraception Study. Contraception.

[75] Song J., Jin Z., Han H., Li M., Guo Y., Guo H., Guo W., He J. (2019). Hormone Replacement Therapies, Oral Contraceptives, Reproductive Factors and Colorectal Adenoma Risk: A Systematic Review and Dose-Response Meta-Analysis of Observational Studies. Colorectal Dis..

[76] Nichols H.B., Trentham-Dietz A., Hampton J.M., Newcomb P.A. (2005). Oral Contraceptive Use, Reproductive Factors, and Colorectal Cancer Risk-Findings from Wisconsin. Cancer Epidemiol. Biomark. Prev..

[77] McMichael A.J., Potter J.D. (1980). Reproduction, Endogenous and Exogenous Sex Hormones, and Colon Cancer—A Review and Hypothesis. J. Natl. Cancer Inst..

[78] Kampman E., Potter J.D., Slattery M.L., Caan B.J., Edwards S. (1997). Hormone Replacement Therapy, Reproductive History, and Colon Cancer: A multicenter, Case-Control Study in the United States. Cancer Causes Control.

[79] Terry M.B., Neugut A.I., Bostick R.M., Sandler R.S., Haile R.W., Jacobson J.S., Fenoglio-Preiser C.M., Potter J.D. (2002). Risk Factors for Advanced Colorectal Adenomas: A Pooled Analysis. Cancer Epidemiol. Biomark. Prev..

[80] Nakhostin L., Stadler A., Stute P. (2021). Impact of Menopausal Hormone Therapy on Colorectal Cancer Risk—A Systematic Review. Clin. Endocrinol. (Oxf).

[81] Kabat G.C., Miller A.B., Rohan T.E. (2008). Oral Contraceptive Use, Hormone Replacement Therapy, Reproductive History and Risk of Colorectal Cancer in Women. Int. J. Cancer.

[82] Ritenbaugh C., Stanford J.L., Wu L., Shikany J.M., Schoen R.E., Stefanick M.L., Taylor V., Garland C., Frank G., Lane D. (2008). Conjugated Equine Estrogens and Colorectal Cancer Incidence and Survival: The Women’s Health Initiative Randomized Clinical Trial. Cancer Epidemiol. Biomark. Prev..

[83] Papaxoinis K., Triantafyllou K., Sasco A.J., Nicolopoulou-Stamati P., Ladas S.D. (2010). Subsite-Specific Differences of Estrogen Receptor Beta Expression in the Normal Colonic Epithelium: Implications for Carcinogenesis and Colorectal Cancer Epidemiology. Eur. J. Gastroenterol. Hepatol..

[84] Barzi A., Lenz A.M., Labonte M.J., Lenz H.J. (2013). Molecular Pathways: Estrogen Pathway in Colorectal Cancer. Clin. Cancer Res..

[85] Chen J., Iverson D. (2012). Estrogen in Obesity-Associated Colon Cancer: Friend or Foe? Protecting Postmenopausal Women but Promoting Late-Stage Colon Cancer. Cancer Causes Control.

[86] Murphy N., Xu L., Zervoudakis A., Xue X., Kabat G., Rohan T.E., Wassertheil-Smoller S., O’Sullivan M.J., Thomson C., Messina C. (2017). Reproductive and Menstrual Factors and Colorectal Cancer Incidence in the Women’s Health Initiative Observational Study. Br. J. Cancer.

[87] Arem H., Park Y., Felix A.S., Zervoudakis A., Brinton L.A., Matthews C.E., Gunter M.J. (2015). Reproductive and Hormonal Factors and Mortality among Women with Colorectal Cancer in the NIH-AARP Diet and Health Study. Br. J. Cancer.

[88] Martinez M.E., Grodstein F., Giovannucci E., Colditz G.A., Speizer F.E., Hennekens C., Rosner B., Willett W.C., Stampfer M.J. (1997). A Prospective Study of Reproductive Factors, Oral Contraceptive Use, and Risk of Colorectal Cancer. Cancer Epidemiol. Biomark. Prev..

[89] Jacobsen B.K., Vollset S.E., Kvale G. (1995). Do Reproductive Factors Influence Colorectal Cancer Survival?. J. Clin. Epidemiol..

[90] Zervoudakis A., Strickler H.D., Park Y., Xue X., Hollenbeck A., Schatzkin A., Gunter M.J. (2011). Reproductive History and Risk of Colorectal Cancer in Postmenopausal Women. J. Natl. Cancer Inst..

[91] Wernli K.J., Wang Y., Zheng Y., Potter J.D., Newcomb P.A. (2009). The Relationship between Gravidity and Parity and Colorectal Cancer Risk. J. Women’s Health.

[92] Luo G., Zhang Y., Wang L., Huang Y., Yu Q., Guo P., Li K. (2016). Risk of Colorectal Cancer with Hysterectomy and Oophorectomy: A Systematic Review and Meta-Analysis. Int. J. Surg..

[93] Segelman J., Lindstrom L., Frisell J., Lu Y. (2016). Population-Based Analysis of Colorectal Cancer Risk after Oophorectomy. Br. J. Surg..

[94] Ritvo P., Myers R.E., Paszat L., Serenity M., Perez D.F., Rabeneck L. (2013). Gender Differences in Attitudes Impeding Colorectal Cancer Screening. BMC Public Health.

[95] Courtenay W.H. (2000). Constructions of Masculinity and Their Influence on Men’s Well-Being-a Theory of Gender and Health. Soc. Sci. Med..

[96] Statistics Canada (2019). Primary Health Care Providers. https://www150.statcan.gc.ca/n1/pub/82-625-x/2020001/article/00004-eng.htm.

[97] Brenes G.A., Paskett E.D. (2000). Predictors of Stage of Adoption for Colorectal Cancer Screening. Prev. Med..

[98] Lipkus I.M., Rimer B.K., Lyna P.R., Pradhan A.A., Conaway M., Woods-Powell C.T. (1996). Colorectal Screening Patterns and Perceptions of Risk among African-American Users of a Community Health Center. J. Community Health.

[99] Menees S.B., Inadomi J., Elta G., Korsnes S., Punch M., Aldrich L. (2010). Colorectal Cancer Screening Compliance and Contemplation in Gynecology Patients. J. Women’s Health.

[100] Carlos R.C., Fendrick A.M., Ellis J., Bernstein S.J. (2004). Can Breast and Cervical Cancer Screening Visits Be Used to Enhance Colorectal Cancer Screening?. J. Am. Coll. Radiol..

[101] McGregor S.E., Bryant H.E. (2005). Predictors of Colorectal Cancer Screening: A Comparison of Men and Women. Can. J. Gastroenterol..

[102] Beydoun H.A., Beydoun M.A. (2008). Predictors of Colorectal Cancer Screening Behaviors Among Average-Risk Older Adults in the United States. Cancer Causes Control.

[103] Rezende L.F.M., Sa T.H., Markozannes G., Rey-Lopez J.P., Lee I.M., Tsilidis K.K., Ioannidis J.P.A., Eluf-Neto J. (2018). Physical Activity and Cancer: An Umbrella Review of the Literature Including 22 Major Anatomical Sites and 770,000 Cancer Cases. Br. J. Sports Med..

[104] Boyle T., Keegel T., Bull F., Heyworth J., Fritschi L. (2012). Physical Activity and Risks of Proximal and Distal Colon Cancers: A Systematic Review and Meta-Analysis. J. Natl. Cancer Inst..

[105] Mahmood S., MacInnis R.J., English D.R., Karahalios A., Lynch B.M. (2017). Domain-Specific Physical Activity and Sedentary Behaviour in Relation to Colon and Rectal Cancer Risk: A Systematic Review and Meta-Analysis. Int. J. Epidemiol..

[106] Shi Y., Li T., Wang Y., Zhou L., Qin Q., Yin J., Wei S., Liu L., Nie S. (2015). Household Physical Activity and Cancer Risk: A Systematic Review and Dose-Response Meta-Analysis of Epidemiological Studies. Sci. Rep..

[107] Ainsworth B.E. (2000). Issues in the Assessment of Physical Activity in Women. Res. Q. Exerc. Sport.

[108] Phongsavan P., Merom D., Marshall A., Bauman A. (2004). Estimating Physical Activity Level: The Role of Domestic Activities. J. Epidemiol. Community Health.

[109] Murphy M.H., Donnelly P., Breslin G., Shibli S., Nevill A.M. (2013). Does Doing Housework Keep You Healthy? The Contribution of Domestic Physical Activity to Meeting Current Recommendations for Health. BMC Public Health.

[110] Schor J.B. (1992). The Overworked American.

[111] Lee W., Hotopf M. (2012). Chapter 10—Critical Appraisal: Reviewing Scientific Evidence and Reading Academic Papers. Core Psychiatry.

[112] Subar A.F., Thompson F.E., Kipnis V., Midthune D., Hurwitz P., McNutt S., McIntosh A., Rosenfeld S. (2001). Comparative Validation of the Block, Willett, and National Cancer Institute Food Frequency Questionnaires-The Eating at America’s Table Study. Am. J. Epidemiol..

[113] Adams S.A., Matthews C.E., Ebbeling C.B., Moore C.G., Cunningham J.E., Fulton J., Hebert J.R. (2005). The Effect of Social Desirability and Social Approval on Self-Reports of Physical Activity. Am. J. Epidemiol..

[114] Du H., Li L., Whitlock G., Bennett D., Guo Y., Bian Z., Chen J., Sherliker P., Huang Y., Zhang N. (2014). Patterns and Socio-demographic Correlates of Domain-Specific Physical Activities and Their Associations with Adiposity in the China Kadoorie Biobank Study. BMC Public Health.

[115] Hebert J.R., Clemow L., Pbert L., Ockenet I.S., Ockene J.K. (1995). Social Desirability Bias in Dietary Self-Report May Compromise the Validity of Dietary Intake Measures. Int. J. Epidemiol..

[116] Viner B., Barberio A.M., Haig T.R., Friedenreich C.M., Brenner D.R. (2019). The Individual and Combined Effects of Alcohol Consumption and Cigarette Smoking on Site-Specific Cancer Risk in a Prospective Cohort of 26,607 adults: Results from Alberta’s Tomorrow Project. Cancer Causes Control.

[117] National Cancer Institute Dietary Assessment Primer. https://dietassessmentprimer.cancer.gov/profiles/record/.

[118] Government of Alberta (2011). Demographic Spotlight-The Visible Minority Population: Recent Trends in Alberta and Canada.

